# Diagnosis and management of neutropenia

**DOI:** 10.1007/s44313-025-00079-1

**Published:** 2025-05-26

**Authors:** Kyoung Il Min, Seonggyu Byeon

**Affiliations:** College of Medicine, Catholic Hematology Hospital, Seoul St. Mary’s Hospital, The Catholic University of Korea, 222 Banpo-daero, Seocho-Gu, Seoul, 06591 Republic of Korea

**Keywords:** Neutropenia, Autoimmune neutropenia, G-CSF, Chemotherapy-induced neutropenia

## Abstract

**Purpose:**

Neutropenia is a hematologic condition characterized by an absolute neutrophil count < 1500/μL, associated with increased infection risk. This review aimed to provide an updated overview of the classification, pathophysiology, etiology, diagnosis, and management of neutropenia in congenital and acquired forms.

**Methods:**

We conducted a comprehensive literature review of various causes of neutropenia, including genetic syndromes, autoimmune disorders, infections, and drug-induced mechanisms. Emphasis was placed on clinical manifestations, underlying mechanisms, diagnostic algorithms, and therapeutic approaches, including recent advances in molecular diagnostics and biologic therapies.

**Results:**

Neutropenia can result from decreased neutrophil production, immune-mediated destruction, or abnormal distribution. Congenital neutropenia is often linked to mutations in genes such as ELANE, HAX1, and SBDS. Acquired neutropenia can be caused by chemotherapy, infections, autoimmune diseases, or nutritional deficiencies. Diagnostic evaluation requires a stepwise approach incorporating clinical history, blood counts, peripheral smear, bone marrow biopsy, and molecular or serologic testing. Treatment depends on the etiology and severity and includes granulocyte colony-stimulating factor, immunosuppressants, antimicrobial prophylaxis, and hematopoietic stem cell transplantation in selected cases.

**Conclusion:**

Neutropenia is a multifactorial disorder requiring individualized evaluation and management. Advances in genetic and immunological diagnostics combined with targeted therapies have improved risk stratification and outcomes. Early recognition and a multidisciplinary approach are essential to reduce infection-related morbidity and prevent progression to hematologic malignancies in high-risk patients.

## Introduction

Neutrophils constitute approximately 60% of the circulating white blood cells and play a vital role in the innate immune system by phagocytizing and destroying pathogens. Neutropenia is defined as an absolute neutrophil count (ANC) below 1500 cells/μL. It can be transient or chronic and mild to severe, and its etiology encompasses a broad spectrum of genetic, autoimmune, infectious, and drug-induced factors. Although mild neutropenia (ANC 1000–1500/μL) is often asymptomatic, severe neutropenia (ANC < 500/μL) is associated with a significantly increased risk for life-threatening infections [1]. The timely identification of the underlying causes is critical for guiding treatment and mitigating infection-related morbidity and mortality.

The prevalence of neutropenia varies according to the etiology, patient population, and geographic factors. The overall prevalence of neutropenia in a large Danish cohort of 373,803 individuals was approximately 1%; acute neutropenia was present in 2.0% and chronic neutropenia in only 0.1% of cases [2]. In contrast, Hsieh et al. reported a higher prevalence of neutropenia in the U.S. population based on data from the 1999 to 2004 National Health and Nutrition Examination Survey. The prevalence of neutropenia (defined as ANC < 1.5 × 10⁹/L) was 4.5% among Black Americans, 0.79% among White Americans, and 0.38% among Mexican-Americans [3]. This disparity may be partially explained by benign ethnic neutropenia, which is observed in 25–50% of African descent and certain Middle Eastern populations [4]. In these cases, chronically low ANC does not predispose individuals to an increased risk of infection. Severe congenital neutropenia (SCN) is a rare genetic disorder characterized by low ANC and recurrent bacterial infections. The incidence of SCNs is approximately 1–2 cases/million live births [5]. In clinical settings, chemotherapy-induced neutropenia is among the most common causes, occurring in approximately 16–50% of patients with cancer receiving cytotoxic chemotherapy, particularly those treated for hematologic malignancies [6]. Given the wide spectrum of causes and clinical presentations of neutropenia, its epidemiology underscores the importance of precise diagnosis and individualized management strategies to reduce infection-related morbidity and mortality.

This review summarizes the current understanding of neutropenia, including its classification, pathophysiology, causes, diagnostic approaches, and management strategies. By integrating insights from recent studies, we aimed to provide a comprehensive resource for clinicians and researchers involved in the diagnosis and treatment of neutropenia.

### Pathophysiology of neutropenia

The development and survival of neutrophils involve a complex process regulated by granulocyte colony-stimulating factor (G-CSF) and various transcription factors. Neutrophils are produced in the bone marrow through a process known as granulopoiesis, which involves the differentiation of myeloid progenitor cells into mature neutrophils. Neutrophils are then released into the circulation, where they have a half-life of approximately 6–8 h before undergoing apoptosis or sequestration in the spleen. Neutropenia is known to result from abnormalities in the production, distribution, and destruction of neutrophils.

#### Reduced production

The most common cause of neutropenia is impaired neutrophil production in the bone marrow, which occurs because of bone marrow failure, genetic mutations, or exposure to myelosuppressive agents. In congenital neutropenic syndromes, such as SCN and cyclic neutropenia, mutations in genes such as ELANE (encoding neutrophil elastase), HAX1, and GFI1 lead to defective neutrophil maturation, resulting in a near-complete absence of mature neutrophils in the circulation [7, 8]. Similarly, Shwachman-Diamond syndrome (SDS) and dyskeratosis congenita impair hematopoietic stem cell function, leading to multilineage cytopenias, including neutropenia [9].

Under acquired conditions, chemotherapy and radiation therapy directly damage the rapidly dividing bone marrow cells, leading to chemotherapy-induced neutropenia. Additionally, viral infections (e.g., Epstein-Barr virus, human immunodeficiency virus, and hepatitis viruses) can transiently suppress bone marrow function, leading to reduced neutrophil output [10–12]. Nutritional deficiencies, particularly those of vitamin B12, folate, and copper, can impair neutrophil production by disrupting DNA synthesis and bone marrow proliferation, resulting in ineffective hematopoiesis [13].

#### Increased destruction

Neutropenia can also result from excessive destruction of circulating neutrophils, often due to immune-mediated mechanisms or excessive activation of the inflammatory response. Autoimmune neutropenia (AIN) occurs when autoantibodies target neutrophils, leading to their premature destruction. This process is common in infants (primary AIN) and adults with systemic autoimmune diseases such as systemic lupus erythematosus (SLE), rheumatoid arthritis (RA) (Felty syndrome), and large granular lymphocyte (LGL) leukemia [14, 15]. Drug-induced neutropenia can also occur through immune-mediated destruction, particularly with medications such as clozapine, sulfasalazine, and antithyroid drugs, which trigger antibody formation against neutrophils. In severe bacterial infections and sepsis, neutrophils undergo excessive activation and apoptosis, leading to bone marrow exhaustion and transient neutropenia. In paroxysmal nocturnal hemoglobinuria and other complement-mediated disorders, neutrophil destruction is exacerbated by dysregulated immune responses [16].

#### Abnormal distribution or sequestration

In some cases, neutropenia arises owing to abnormal neutrophil trafficking and sequestration, rather than intrinsic defects in production or destruction. Hypersplenism, observed in liver cirrhosis, lymphoma, and chronic infections such as malaria, leads to excessive sequestration of neutrophils within the enlarged spleen, resulting in a functional reduction in circulating neutrophils [17, 18]. In conditions such as bone marrow fibrosis and myelophthisis (bone marrow infiltration by malignancies such as leukemia and metastatic cancer), neutrophils are unable to exit the bone marrow into the circulation, contributing to peripheral neutropenia. Certain genetic disorders, such as myelokathexis in warts, hypogammaglobulinemia, infections, and myelokathexis (WHIM) syndrome, lead to the retention of neutrophils in the bone marrow owing to defective CXCR4 signaling, preventing their release into the peripheral blood [19].

### Clinical implications of neutropenia pathophysiology

The underlying mechanism of neutropenia determines its clinical severity and management approaches. Decreased neutrophil production (e.g., congenital and chemotherapy-induced neutropenia) often requires growth factor support (G-CSF) or bone marrow transplantation. In contrast, immune-mediated neutropenia (e.g., AIN and drug-induced neutropenia) may respond to immunosuppressive therapy or withdrawal of the offending agent. Neutropenia due to sequestration (e.g., hypersplenism) may improve with treatment of the underlying disorder (e.g., splenectomy). A systematic diagnostic approach, including bone marrow biopsy, genetic testing, and immune profiling, is essential for determining the underlying mechanisms and guiding appropriate treatment strategies.

### Classification of neutropenia and management

Neutropenia can be classified based on severity, duration, and etiology (Table 1). Each classification method provides critical insight into disease progression and treatment approaches.

**Table 1:** Classification of neutropenia

Classification Domain	Subtypes	Definition/Description
** Severity**	Mild	ANC 1.0rityDescr’/L
	Moderate	ANC 0.5eityDescr’/L
	Severe	ANC < 0.5 yDes’/L
** Duration**	Acute	Lasts < 3 months
	Chronic	Lasts > 3 months
** Etiology**	Congenital	Genetic causes (e.g., SCN, cyclic neutropenia, SDS)
	Acquired	Due to external factors (e.g., infections, medications, autoimmune diseases)
** Mechanism**	Decreased production	Bone marrow failure, myelodysplastic syndrome, chemotherapy
	Increased destruction	Autoimmune neutropenia, drug-induced neutropenia
	Marginalization/sequestration	Hypersplenism, hemodialysis
** Cause**	Infections	Viral (e.g., EBV, HIV), bacterial, parasitic
	Drug-induced	Chemotherapy, antibiotics, psychotropic drugs
	Immune-mediated	Autoimmune neutropenia, Felty syndrome
	Hematologic disorders	Leukemia, myelodysplastic syndrome, aplastic anemia
	Nutritional deficiencies	Vitamin B12, folate, copper deficiency
** Associated Conditions**	Pancytopenia	Associated with bone marrow failure and hematologic malignancies
	Isolated neutropenia	No involvement of other cell lineages

The severity of neutropenia is divided into mild, moderate, and severe depending on ANC levels. Mild neutropenia (ANC 1000–1500/μL) poses minimal infection risk, often resolving without intervention. Moderate neutropenia (ANC between 500 and 1000/μL) increases susceptibility to infections, particularly affecting the skin and mucous membranes. Severe neutropenia is defined as an ANC < 500/μL, which significantly raises the likelihood of opportunistic infections, including bacterial and fungal bloodstream infections, pneumonia, and severe mucosal ulcerations. Patients with severe neutropenia are at high risk of febrile neutropenia, a medical emergency requiring urgent antibiotic therapy and supportive care [1].

Classification by duration differentiates acute and chronic neutropenia. While acute neutropenia may be self-limiting, chronic neutropenia warrants a more extensive investigation to rule out malignancies or hereditary causes. Acute neutropenia lasting less than three months is often caused by transient factors such as viral infections, drug reactions, or temporary bone marrow suppression. Acute neutropenia generally resolves once the underlying condition is treated or is self-limiting. Chronic neutropenia persists for more than three months and is commonly associated with congenital disorders, autoimmune diseases, or hematologic malignancies. Chronic forms require a more in-depth diagnostic approach to identify the potential underlying causes that may require long-term management.

### Congenital neutropenia

Congenital neutropenia includes SCN, cyclic neutropenia, SDS, and Chediak-Higashi syndrome, all of which result from genetic mutations that affect neutrophil production or function [20, 21].

SCN is a well-characterized form of congenital neutropenia. It is mainly caused by autosomal dominant mutations (up to 60%) in the *ELANE* gene, which encodes neutrophil elastase. Misfolding of neutrophil elastase proteins leads to the activation of the unfolded protein response and apoptosis of myeloid precursors in the bone marrow. Other genes implicated in SCN include *HAX1*, *GFI1*, and *WAS*, which regulate apoptosis, transcriptional activity, and cytoskeletal function of neutrophils [21–23]. Patients with SCN present with recurrent bacterial infections, including cellulitis, pneumonia, and sepsis, during early infancy. Bone marrow biopsy typically reveals maturation arrest at the promyelocytic or myelocytic stage.

Cyclic neutropenia is a rare autosomal dominant disorder caused by specific mutations in the *ELANE* gene [24]. It is characterized by regular oscillations in neutrophil count that occur approximately every 21 days. The nadir period of neutropenia typically lasts 3–5 days, during which patients are at an increased risk of infection. The symptoms include recurrent fever, oral ulcers, and skin infections. Despite its periodic nature, cyclic neutropenia is usually less severe than SCN, and most patients respond well to G-CSF therapy.

SDS is another congenital form of neutropenia caused by mutations in the *SBDS* gene, which regulates ribosomal function and cell stress responses [9]. Patients with SDS often present with neutropenia, exocrine pancreatic insufficiency, and skeletal abnormalities. Neutropenia in SDS is due to increased apoptosis of myeloid precursors in the bone marrow. Approximately 30% of patients with SDS develop myelodysplastic syndrome (MDS) or acute myeloid leukemia (AML) during adolescence or early adulthood.

Long-term G-CSF therapy is the mainstay of treatment in patients with congenital neutropenia. G-CSF significantly reduces the risk of life-threatening infections and improves survival in these patients. However, SCN carries a 10–30% lifetime risk of progression to hematologic malignancies such as MDS or AML, necessitating regular monitoring of bone marrow function and cytogenetic changes. In cases in which patients become refractory to G-CSF or show signs of leukemic transformation, hematopoietic stem cell transplantation (HSCT) is the only curative option. HSCT is also indicated in severe bone marrow failure syndromes, such as SDS, where neutropenia is part of a broader spectrum of hematopoietic dysfunction. In cyclic neutropenia, G-CSF therapy reduces the frequency and severity of neutropenic episodes, thus lowering the risk of infection in certain cases.

### Acquired neutropenia

Acquired neutropenia is more common than congenital forms and can result from various factors.

Drug-induced neutropenia is a relatively frequent cause with an estimated incidence ranging from 2.4 to 15.4 cases per million population [25–27]. The mechanism of drug-induced neutropenia often involves immune-mediated destruction through drug-specific antibodies or direct toxicity to myeloid precursors in the bone marrow (Table 2). Commonly implicated drugs include antibiotics (e.g., penicillins and sulfonamides), antipsychotics (e.g., clozapine), antithyroid drugs (e.g., methimazole and propylthiouracil), and chemotherapeutic agents (e.g., cyclophosphamide and methotrexate). Clozapine-induced neutropenia, which affects approximately 1% of treated patients, is one of the most well-characterized forms, with onset typically occurring within the first three months of treatment. In cases of drug-induced neutropenia, the discontinuation of the offending drug often leads to recovery. If neutropenia persists or is associated with an infection, G-CSF therapy may be considered. For chemotherapy-induced neutropenia, one of the most common causes of severe neutropenia, the primary management strategy is the use of G-CSF, such as filgrastim or pegfilgrastim, which stimulates the production and release of neutrophils from the bone marrow. G-CSF is recommended for patients at high risk of febrile neutropenia, including those receiving intensive chemotherapy for leukemia, lymphoma, or solid tumors, as well as those with pre-existing bone marrow suppression. G-CSF has been shown to reduce infection-related morbidity, hospitalization, and chemotherapy dose delays, thereby improving the overall treatment outcomes. However, routine prophylactic use of G-CSF in all patients with cancer is not universally recommended, as the benefits must be weighed against potential adverse effects, such as bone pain, leukocytosis, and, in rare cases, splenic rupture [28, 29].

**Table 2:** Drug-induced neutropenia causes

Drug Class	Examples	Mechanism of Action
**Antibiotics**	Penicillins, Sulfonamides, Cephalosporins, Vancomycin, Cotrimoxazole, Metronidazole	Direct toxicity to bone marrow, immune-mediated destruction
**Antipsychotics**	Clozapine, Chlorpromazine, Olanzapine	Direct bone marrow suppression, immune-mediated destruction
**Antithyroid drugs**	Propylthiouracil, Methimazole, Carbimazole	Immune-mediated destruction of neutrophils
**Anticonvulsants**	Carbamazepine, Phenytoin, Valproate	Bone marrow suppression, immune-mediated destruction
**Chemotherapy**	Cyclophosphamide, Methotrexate, Cisplatin	Direct toxicity to myeloid precursors
**Anti-inflammatory drugs**	NSAIDs (Indomethacin, Diclofenac)	Immune-mediated destruction, bone marrow suppression
**Cardiovascular drugs**	Hydralazine, Procainamide, Quinidine	Immune-mediated destruction
**Immunosuppressants**	Tacrolimus, Cyclosporine, Azathioprine	Bone marrow suppression, direct toxicity to myeloid precursors
**Other**	Dipyrone, Ticlopidine, Rituximab, Deferiprone	Immune-mediated destruction, direct toxicity to neutrophil precursors

Viral infections are a well-recognized cause of neutropenia, often leading to transient decreases in neutrophil count through direct bone marrow suppression or immune-mediated destruction. EBV, HIV, hepatitis viruses, and parvovirus B19 are among the most common viral agents implicated in neutropenia [10–12]. EBV infection can cause severe neutropenia through direct infection of myeloid progenitor cells or the induction of anti-neutrophil antibodies. HIV-associated neutropenia is associated with bone marrow suppression and increased peripheral neutrophil destruction, which are often exacerbated by concurrent infection and drug toxicity. Cytomegalovirus and parvovirus B19 have been shown to suppress granulopoiesis by infecting the bone marrow stromal cells. While most cases of virus-induced neutropenia are self-limiting, severe or prolonged neutropenia may require antiviral therapy and supportive care, including the use of G-CSF to restore neutrophil levels and reduce the risk of secondary infections.

AIN occurs when autoantibodies target neutrophils, which leads to increased peripheral destruction and impaired bone marrow production. AIN can occur in isolation (primary AIN) or in association with systemic autoimmune diseases, such as SLE or RA [14, 30]. Patients with AIN often present with recurrent infections of the skin and respiratory tract; however, the severity of infections varies depending on the ANC level. Treatment is often tailored to the severity of neutropenia and the presence of infection. Primary AIN in children is usually benign and self-limiting**,** with most cases resolving within 2–3 years without the need for intervention. In contrast, secondary AIN, which is associated with systemic disease, may require immunosuppressive therapy with corticosteroids, intravenous immunoglobulin, or rituximab, depending on the disease severity. Methotrexate and cyclophosphamide have also been used to treat refractory cases of LGL leukemia-associated neutropenia. G-CSF may be considered when patients experience recurrent infections or profound neutropenia (ANC < 200/µL), although its efficacy varies.

LGL leukemia is suspected based on increased LGLs in peripheral blood, typically > 2 × 10⁹/L. Flow cytometry confirms the diagnosis by identifying CD3 +, CD8 +, CD57 +, and CD16 + phenotype in T-cell LGL leukemia, with possible loss of CD5 or CD7. T-cell receptor clonality testing confirms clonal expansion, supporting this diagnosis. Bone marrow biopsy is not always required but may show infiltration of CD8 + and CD57 + cells. Approximately 40% of cases harbor STAT3 mutations, which assist in diagnosis and prognosis [15, 31, 32]. Treatment of LGL leukemia-associated neutropenia typically involves immunosuppressive agents such as methotrexate, cyclosporine, or oral cyclophosphamide to reduce clonal lymphocyte proliferation and improve neutrophil counts. G-CSF is often used to stimulate neutrophil production and reduce the risk of infection in patients with severe neutropenia or recurrent infections. Corticosteroids may also be considered for refractory cases or when autoimmune complications are present.

Nutritional deficiencies are a well-recognized but often overlooked cause of neutropenia resulting from impaired neutrophil production due to an inadequate supply of essential nutrients required for granulopoiesis. Vitamin B12 and folate deficiencies are the most common nutritional causes, leading to ineffective hematopoiesis and megaloblastic changes in the bone marrow. Copper deficiency can also cause neutropenia by disrupting granulocyte maturation and increasing apoptosis of myeloid precursors. Zinc and iron deficiencies have been implicated in neutropenia because of their roles in immune cell function and DNA synthesis. Many individuals with nutritional deficiency-associated neutropenia may have conditions that predispose them to decreased intake or absorption, such as dietary restriction (e.g., strict vegan diet) or gastrointestinal surgery (e.g., bariatric surgery, gastrectomy, or small bowel resection). These symptoms typically resolve with targeted repletion of deficient nutrients. In cases of severe or persistent neutropenia, adjunctive therapy with G-CSF may accelerate recovery and lower the risk of secondary infections.

### Diagnostic approach

The diagnosis of neutropenia requires a thorough clinical evaluation. Clinical history should assess the onset, duration, and presence of symptoms, such as recurrent infections, fever, or mucosal ulcerations. A complete blood count with differential is the primary diagnostic tool used to assess ANC and identify any accompanying cytopenia or hematologic abnormalities. Peripheral blood smears are useful for evaluating neutrophil morphology and detecting immature forms or blasts, which may indicate bone marrow infiltration or myelodysplasia. Bone marrow aspiration and biopsy are indicated in patients with severe or unexplained neutropenia. In congenital neutropenia, bone marrow examination typically reveals maturation arrest at the promyelocytic or myelocytic stage. By contrast, drug-induced neutropenia or AIN is often associated with a hyperplastic or normocellular bone marrow.

Genetic testing is essential in suspected cases of congenital neutropenia. Mutations in *ELANE*, *HAX1*, *GFI1*, and *SBDS* can confirm the diagnosis of SCN, cyclic neutropenia, or SDS. Serologic tests for anti-neutrophil antibodies can help diagnose AIN. Antibody testing using the granulocyte agglutination test or the granulocyte immunofluorescence test can confirm the presence of anti-neutrophil antibodies. Flow cytometry can be used to evaluate lymphocyte subsets and assess LGL expansion, a characteristic of LGL leukemia. Autoimmune markers, including antinuclear antibodies and rheumatoid factors, should be evaluated in patients with suspected AIN. Nutritional assessment of vitamin B12, folate, and copper deficiencies is essential when dietary insufficiency is suspected.

### Therapeutic approach

The general management of neutropenia depends on its severity, underlying cause, and associated complications**,** with the primary goals being prevention of infection, reversal of the underlying cause, and improvement of neutrophil levels when necessary. Supportive care and monitoring may be sufficient in cases of mild and transient neutropenia, whereas severe or chronic neutropenia often requires targeted interventions, such as growth factor therapy, immunosuppression, or bone marrow transplantation.

The most important step in managing neutropenia is assessing the infection risk, as patients with an ANC < 500/µL are at a significantly increased risk of opportunistic infections, sepsis, and mortality. Febrile neutropenia, defined as fever > 38.3 °C with ANC < 500/µL, is a medical emergency that requires immediate hospitalization, broad-spectrum intravenous antibiotics, and aggressive supportive care. Empirical antibiotic therapy should include coverage against Gram-negative bacilli (e.g., *Pseudomonas aeruginosa*) and Gram-positive organisms (e.g., *Staphylococcus aureus*), typically with agents such as piperacillin-tazobactam, cefepime, meropenem, or imipenem-cilastatin, depending on the local resistance patterns. In cases of persistent fever despite antibiotic therapy, antifungal coverage (e.g., fluconazole, voriconazole, or echinocandins) should be considered, particularly in immunocompromised patients. Patients with prolonged neutropenia and recurrent infections may also benefit from prophylactic antibiotics (e.g., fluoroquinolones) and antifungals to reduce the risk of severe infections [33].

### Prognosis and long-term considerations

The primary determinants of long-term outcomes include the severity of neutropenia, frequency and severity of infections, presence of bone marrow dysfunction, and genetic predisposition to hematologic malignancies. While some cases, such as transient post-infectious or drug-induced neutropenia, have an excellent prognosis with no long-term consequences, others, such as SCN and MDS, carry a high risk of complications, including life-threatening infections and malignant transformations.

In patients with mild or moderate neutropenia (ANC > 1000/µL), particularly in cases of chronic idiopathic neutropenia or AIN, the prognosis is generally favorable. Most individuals remain asymptomatic or experience occasional infections requiring periodic monitoring. In childhood primary AIN, the condition often resolves spontaneously within 2–3 years, with most children maintaining normal immune function until adulthood. However, neutropenia may persist for years in patients with secondary AIN, particularly when associated with SLE, RA, or LGL leukemia, necessitating long-term immunosuppressive therapy and infection prophylaxis.

In patients with congenital neutropenia, long-term outcomes largely depend on specific genetic mutations and the responses to G-CSF therapy. Long-term G-CSF therapy effectively reduced the risk of infection and improved patient survival. However, SCN carries a 10–30% lifetime risk of progression to MDS or AML, particularly in patients requiring high-dose G-CSF for ANC maintenance. Regular bone marrow monitoring with cytogenetic analysis is crucial for detecting the early signs of malignant transformation in these patients. Similarly, patients with SDS or dyskeratosis congenita, both of which affect multiple hematopoietic lineages, are at a high risk of bone marrow failure and MDS, necessitating lifelong surveillance and consideration of HSCT when marrow function declines.

## Conclusion

Neutropenia is a complex and heterogeneous condition with various causes, clinical presentations, and management strategies. It can result from genetic mutations affecting neutrophil maturation (congenital neutropenia), immune-mediated destruction (AIN), infections, drug-induced bone marrow suppression, or systemic conditions, such as hematologic malignancies and bone marrow failure syndromes. The diagnostic approach must be systematic and comprehensive, incorporating clinical history, laboratory testing, bone marrow examination, and genetic analysis, to determine the underlying cause and guide appropriate treatment. Management strategies vary depending on the etiology, ranging from watchful waiting in mild, self-limiting cases to aggressive interventions such as G-CSF therapy or HSCT in severe or high-risk cases. A multidisciplinary approach involving hematologists, infectious disease specialists, genetic counselors, and transplant teams is essential to ensure the best possible prognosis for patients with chronic or high-risk neutropenia. By leveraging early diagnosis, targeted therapies, and innovative research, clinicians can optimize patient care and significantly improve the prognosis of patients with neutropenia.

## Data Availability

No datasets were generated or analysed during the current study.
